# Supplementary material for: The signaling lipid sphingosine 1-phosphate regulates mechanical pain
Source: eLife. 2018 Mar 21;7:e33285. doi: 10.7554/eLife.33285 (PMC5896955; doi:10.7554/eLife.33285)
Supplement: Transparent reporting form [file elife-33285-transrepform.docx]

***eLife’s* transparent reporting form**

We encourage authors to provide detailed information *within their submission* to facilitate the interpretation and replication of experiments. Authors can upload supporting documentation to indicate the use of appropriate reporting guidelines for health-related research (see [EQUATOR Network](http://www.equator-network.org/%20)), life science research (see the [BioSharing Information Resource](https://biosharing.org/)), or the [ARRIVE guidelines](http://www.plosbiology.org/article/info:doi/10.1371/journal.pbio.1000412) for reporting work involving animal research. Where applicable, authors should refer to any relevant reporting standards documents in this form.

If you have any questions, please consult our Journal Policies and/or contact us: [editorial@elifesciences.org](mailto:editorial@elifesciences.org).

**Sample-size estimation**

- You should state whether an appropriate sample size was computed when the study was being designed
- You should state the statistical method of sample size computation and any required assumptions
- If no explicit power analysis was used, you should describe how you decided what sample (replicate) size (number) to use

Sample sizes were not computed beforehand, but sample sizes were determined for in vitro, ex vivo, and in vivo experiments based on similar types of experiments in recent papers in the field.

Please outline where this information can be found within the submission (e.g., sections or figure legends), or explain why this information doesn’t apply to your submission:

**Replicates**

- You should report how often each experiment was performed
- You should include a definition of biological versus technical replication
- The data obtained should be provided and sufficient information should be provided to indicate the number of independent biological and/or technical replicates
- If you encountered any outliers, you should describe how these were handled
- Criteria for exclusion/inclusion of data should be clearly stated
- High-throughput sequence data should be uploaded before submission, with a private link for reviewers provided (these are available from both GEO and ArrayExpress)

Please outline where this information can be found within the submission (e.g., sections or figure legends), or explain why this information doesn’t apply to your submission:

The figure legends provide details on biological replicates (i.e. number of animals, number of fibers, or number of cells, where relevant) for experiments. For all in vivo experiments, N = number of mice. For ex vivo skin nerve recording, n = number of fibers. For in vitro whole cell electrophysiology n = number of cells, whereas for calcium imaging n = number of wells of > 100 cells each. We have also uploaded source data files providing more information on the behavioral experiments, skin nerve recording, and whole cell electrophysiology. For Figure 5a, data used in bar graph were omitted because they are provided in Supplemental Figure 3 as a dot plot. No outliers or data were excluded.

**Statistical reporting**

- Statistical analysis methods should be described and justified
- Raw data should be presented in figures whenever informative to do so (typically when N per group is less than 10)
- For each experiment, you should identify the statistical tests used, exact values of N, definitions of center, methods of multiple test correction, and dispersion and precision measures (e.g., mean, median, SD, SEM, confidence intervals; and, for the major substantive results, a measure of effect size (e.g., Pearson's r, Cohen's d)
- Report exact p-values wherever possible alongside the summary statistics and 95% confidence intervals. These should be reported for all key questions and not only when the p-value is less than 0.05.

Please outline where this information can be found within the submission (e.g., sections or figure legends), or explain why this information doesn’t apply to your submission:

Exact p-values, Ns, and statistical tests, where relevant, are reported in the figure legends. P values less than 0.001 are not reported using exact values. Mean and SEM or SD (where appropriate, indicated in the legends) is shown on the graphs. Data are plotted and analyzed according to standard methods in the pain and touch fields. Details regarding data analysis are reported in the Methods section with relevant citations to previously published studies employing same methods.

(For large datasets, or papers with a very large number of statistical tests, you may upload a single table file with tests, Ns, etc., with reference to sections in the manuscript.)

**Group allocation**

- Indicate how samples were allocated into experimental groups (in the case of clinical studies, please specify allocation to treatment method); if randomization was used, please also state if restricted randomization was applied
- Indicate if masking was used during group allocation, data collection and/or data analysis

Please outline where this information can be found within the submission (e.g., sections or figure legends), or explain why this information doesn’t apply to your submission:

Samples per group were balanced wherever possible, and control groups were included in each experiment, so no randomization was used. All behavioral and ex vivo experiments were performed while blind to experimental condition and/or genotype of animal.

**Additional data files (“source data”)**

- We encourage you to upload relevant additional data files, such as numerical data that are represented as a graph in a figure, or as a summary table
- Where provided, these should be in the most useful format, and they can be uploaded as “Source data” files linked to a main figure or table
- Include model definition files including the full list of parameters used
- Include code used for data analysis (e.g., R, MatLab)
- Avoid stating that data files are “available upon request”

Please indicate the figures or tables for which source data files have been provided:

Figures 1, 2, 5, 6, and 7 have associated source data files. Each sheet in the .xlsx file represents a subfigure, with the exception of Figure 6’s table which lists the details for each fiber in the skin nerve recording experiments.
